# Supplementary figures and images for: Repeatability and Reproducibility of Retinal Fractal Dimension Measured with Swept-Source Optical Coherence Tomography Angiography in Healthy Eyes: A Proof-of-Concept Study
Source: Diagnostics (Basel). 2022 Jul 21;12(7):1769. doi: 10.3390/diagnostics12071769 (PMC9323583; doi:10.3390/diagnostics12071769)

Supplementary Materials:

**Figure S1.** Skeleton—Red.

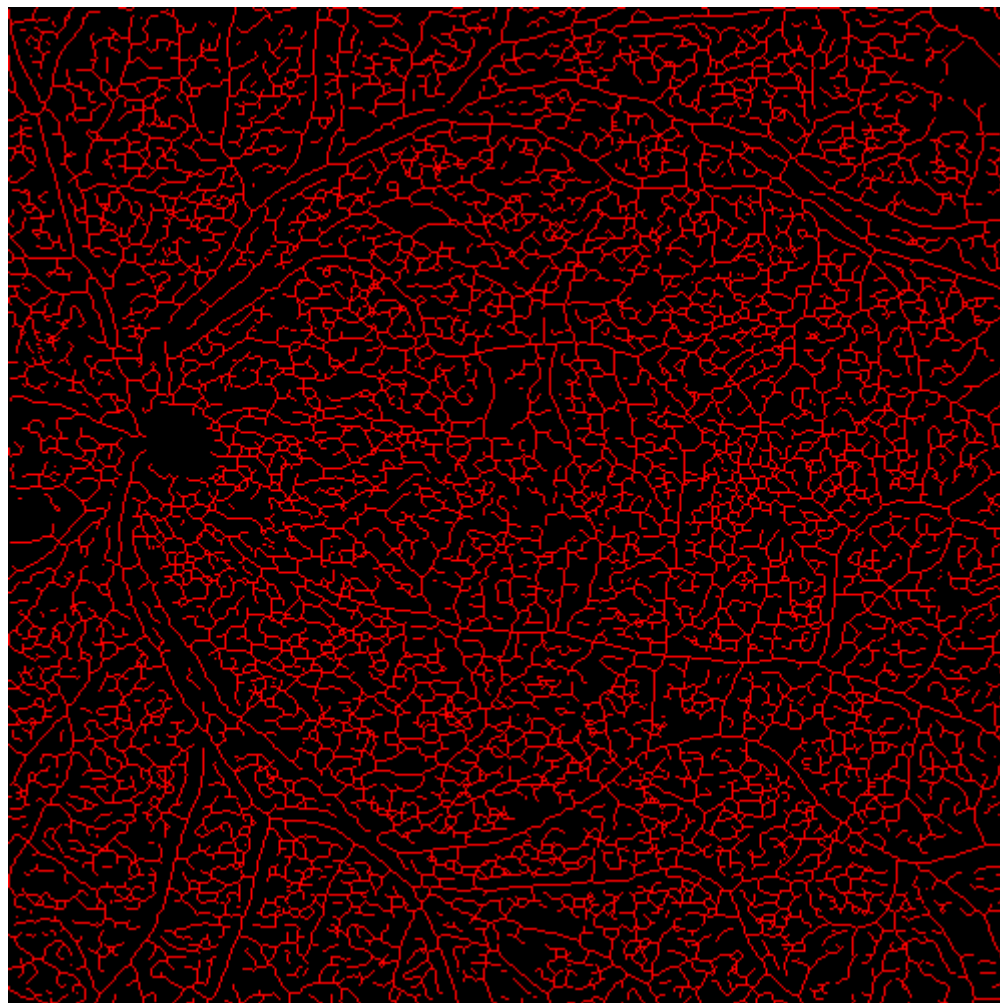

**Figure S2.** Skeleton superimposed on Greyscale.

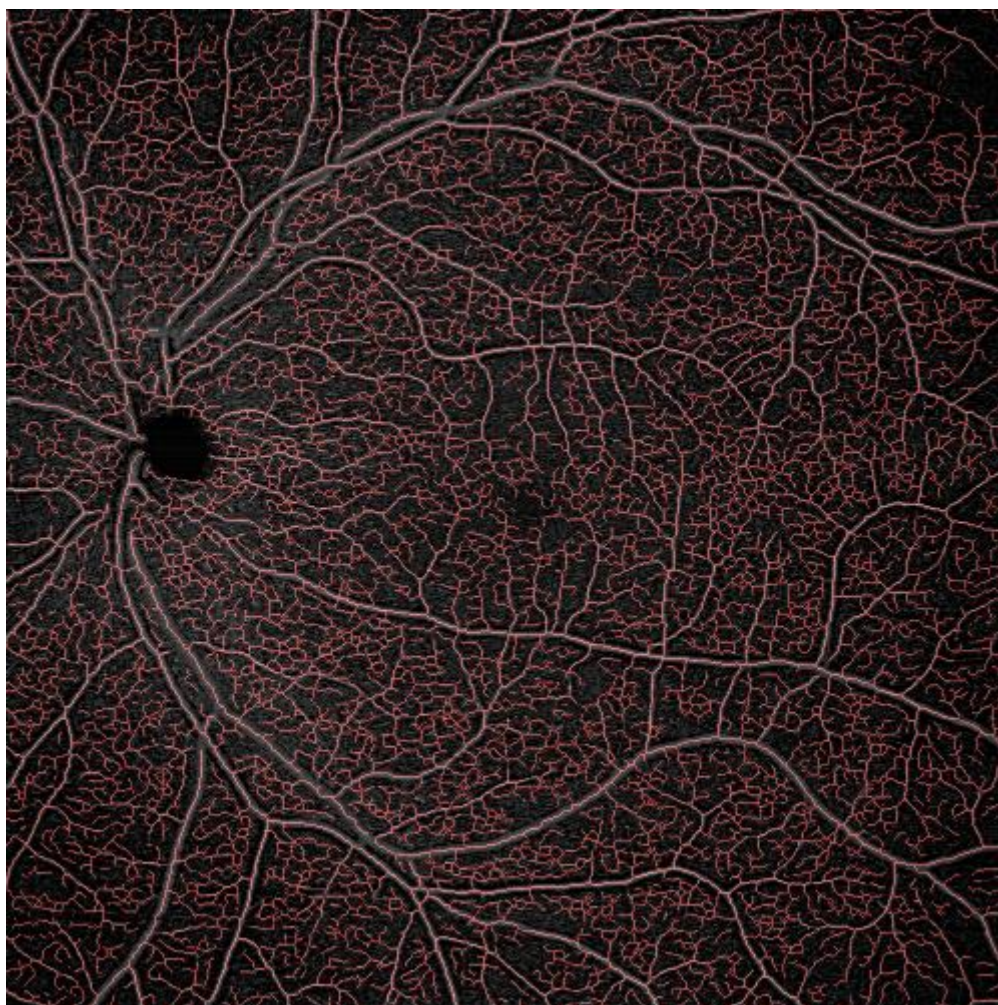

Supplement: Supplementary file 1 [file diagnostics-12-01769-s001.zip › diagnostics-1787867-supplementary.pdf]
